# When wuthering winds create fluttering fields: structural and biomechanical properties determine canopy light fluctuation properties of 10 wheat cultivars

**DOI:** 10.1111/nph.70975

**Published:** 2026-02-05

**Authors:** Maxime Durand, Jonathon A. Gibbs, Erik H. Murchie, T. Matthew Robson, Alexandra J. Gibbs

**Affiliations:** ^1^ Université Paris‐Saclay CNRS, AgroParisTech, Ecologie Société Evolution Gif‐sur‐Yvette 91190 France; ^2^ Faculty of Biological and Environmental Sciences, Organismal and Evolutionary Biology (OEB), Viikki Plant Science Centre (ViPS) University of Helsinki Helsinki 00014 Finland; ^3^ Agriculture and Environmental Sciences, School of Biosciences University of Nottingham Sutton Bonington Campus Loughborough LE12 5RD UK; ^4^ Division of Plant and Crop Science, School of Biosciences University of Nottingham Sutton Bonington Campus Loughborough LE12 5RD UK; ^5^ National School of Forestry, Institute of Science & Environment University of Cumbria Ambleside LA22 9BB UK

**Keywords:** biomechanics, light, movement, photosynthesis, wheat (*Triticum aestivum*), windflecks

## Abstract

- Wind‐driven plant movement generates rapid light fluctuations (windflecks), which can impact canopy photosynthesis. Targeting crop photosynthesis in dynamic light provides a potential path towards boosting yield. Here, we quantified how plant architecture and biomechanics modulate such windflecks across 10 high‐yielding cultivars of winter wheat (*Triticum aestivum*).
- Using synchronized high‐frequency measurements of irradiance, wind speed, and canopy motion (quantified by frame differencing from video), we assessed the propensity of wheat cultivars to move (motion sensitivity), and the ability for movement to produce windflecks (light modulation efficiency) in the field.
- There was up to 10‐fold variation in the quantity of motion between cultivars under identical wind speeds. Cultivars also exhibited structural trade‐offs and specific *in canopy* windfleck properties. Some had low motion under wind but produced frequent windflecks when moving, whereas others exhibited high motion under similar wind but varied in windfleck frequency. Overall, windfleck properties were best explained by aerodynamic traits: cultivars with narrower leaves and lower leaf‐to‐stem mass ratios were associated with more intense windflecks.
- These findings establish that wheat cultivars actively modulate their light environment through biomechanical traits. By integrating plant motion into crop models, favouring motion–light relationships, which could provide a critical route to yield improvements in turbulent environments.

## Introduction

Leaf photosynthesis has been predominantly assessed for its steady state performance under constant light (Kaiser *et al*., 2018). Yet, current research on dynamic photosynthesis, ubiquitous to field conditions, has drawn attention to an opportunity to improve yield in major crops such as *Triticum aestivum* (wheat; Salter *et al*., 2019), *Oryza sativa* (rice; Acevedo‐Siaca *et al*., 2020), *Glycine max* (soybean; De Souza *et al*., 2022), and *Nicotiana tabacum* (tobacco; Kromdijk *et al*., 2016).

In all crops, light fluctuations during the day are mainly a result of clouds, solar movement and the wind, which operate at different time scales. Cloudflecks (i.e. fluctuations in light due to clouds alone at the top part of the canopy) and sunflecks created by solar movement (i.e. fluctuations caused by shifting occlusion patterns within the canopy) typically last between a few minutes to hours (Sellaro *et al*., 2024) and are likely to induce closing then reopening of the stomata to regulate the diffusion of CO_2_ in the leaf. During cloudflecks and sunflecks, the relaxation of nonphotochemical quenching (NPQ), the process of dissipating excess light energy as heat, will be crucial to optimize CO_2_ assimilation at low light and prevent damage to photosynthetic machinery (Kromdijk *et al*., 2016; De Souza *et al*., 2022). Repeated and/or persistent cloud/sun flecks may also affect longer‐term photosynthetic performance by reducing the ribulose‐1,5‐bisphosphate carboxylase–oxygenase (Rubisco) activation state (Taylor *et al*., 2022).

By contrast, wind‐induced movement in crops, otherwise termed mechanical canopy excitation (Burgess *et al*., 2016), produces fluctuations called ‘windflecks’, which generally occur at much greater frequency (Pearcy *et al*., 1990; Burgess *et al*., 2021; Durand & Robson, 2023). Despite being ubiquitous in all field environments, wind and the corresponding fluctuations in light which it produces are rarely considered in terms of their impacts on biomass production and yield.

The overwhelming majority of computer models describing plant structure and functions are static, and do not account for movements of stems and leaves which will impact absorbed radiation, leaf temperatures, and gas exchange. Indeed, the light environment within a crop canopy is scarcely ever constant, with fluctuations between sun and shade being the norm. Even if one assumes an instantaneous response of photosynthesis to the light environment, as do nearly all models of photosynthesis currently in use (Slattery *et al*., 2018), steady light will allow more CO_2_ to be assimilated than fluctuating light of the same average irradiance. This is because the gain in carbon uptake during the high part of the fluctuation will always be less than the loss at low light due to the asymptotic shape of the photosynthetic light response.

The time needed for plants to adjust dynamically to their environment limits CO_2_ assimilation further. Long *et al*. (2022) calculated that sun‐to‐shade transitions may reduce the potential CO_2_ assimilable by crops by up to 40% for a canopy over a day when compared to an instantaneous response. These inefficiencies present a unique opportunity to improve crop photosynthesis (Lawson *et al*., 2012; Ort *et al*., 2015; Murchie & Ruban, 2020), because the large diversity in the speed of adjustment between (McAusland *et al*., 2016; Kang *et al*., 2024) and within species (Qu *et al*., 2016; Soleh *et al*., 2016; Salter *et al*., 2019; Burgess *et al*., 2021) allows for the selection of cultivars which track their environment more closely, and thus use the fluctuating light available to them more efficiently.

The effect of wind on light fluctuations within the canopy is difficult to simulate because of the static nature of canopy models (Slattery *et al*., 2018). Yet, taking advantage of advanced imaging and the use of ray tracing, Burgess *et al*. (2016) estimated that wind‐induced movement of leaves can improve daily carbon gain by 7% to 17% via increased light interception depending on wind direction in rice (*Oryza sativa*). Still, before we can harness canopy structure and movement to improve canopy photosynthesis, important steps must first be taken. We still know very little about how the structure and movement of plants create windflecks of different shape, size, and colour (Way & Pearcy, 2012; Burgess *et al*., 2021), yet this knowledge is critical if we are to evaluate how plants themselves dictate the natural fluctuations of sunlight and its resulting impact on carbon gain and yields.

Recent evidence suggests that among crops, those species with stronger windflecks (i.e. a larger resulting increase in irradiance) also displayed higher canopy light transmission and leaf angle, whereas longer‐lasting windflecks tended to be associated with crops of lower plant height and leaf width (LW) (Durand & Robson, 2023). Similar results have been found within a single species, where differences in canopy structure between wheat cultivars lead to differences in windfleck frequency (Burgess *et al*., 2021).

Globally, wheat remains the second most important food source after rice, but its yields are stagnating across Europe and many parts of the globe (Ray *et al*., 2012), which compels us to find new paths of innovation. Thus, combined with established variation in windfleck characteristics, wheat is proposed as a good model crop to investigate the light environment created by within‐species variability in structure and propensity to move in the wind. We collected time series of photosynthetic photon flux density (PPFD) at high frequency to record the rapid light fluctuations induced by wind in 10 contrasting high‐yielding field‐grown wheat cultivars. By associating this data to simultaneous measurements of wind speed and plant movement based on video analysis, we aimed to identify within‐species differences in how wind creates movement in the canopy. In particular, we asked: (1) Do wheat cultivars differ in their motion in response to wind? (2) Is increased motion associated with distinct windfleck patterns? (3) If so, are windfleck properties related to structural traits of the wheat cultivar? As such, we present the first step required to understand how the light environment in plant canopies is dynamically shaped by plant movements under the wind.

## Materials and Methods

### Plant material and growth

High‐yielding *T. aestivum* L. (Winter wheat) cultivars were grown in 2023 as part of a field trial at the University of Nottingham (Sutton Bonington Campus) in Leicestershire (UK, 52.834 N, 1.243 W). Ten cultivars were selected as representative of those commonly grown in the UK, as follows: Crusoe (LG), KWS Extase (KWS), KWS Firefly (KWS), KWS Kinetic (KWS), Mayflower (Elsoms), RGT Wilkinson (RAGT), Skyfall (RAGT), SY Insitor (Syngenta), Theodore (DSV), and Zoom (Elsoms). All had erect leaves (Supporting Information Fig. S1). The soil was a sandy loam, previously used to grow winter oats (*Avena sativa*, L.). Before sowing, the field was ploughed, power harrowed, and rolled after drilling. The experiment had a completely randomized split‐plot design with three replicate blocks including each of the 10 cultivars. Within each block, each cultivar made up a 2 × 1‐m plot separated by 50 cm rows of bare earth in both directions. Seeds were adjusted by cultivar to achieve a common seeding rate of 350 seeds m^−2^ and were sown on 6 October 2022. 120 kg ha^−1^ of ammonium nitrate fertilizer was applied in a two‐split program, and phosphorus (P) and potassium (K) fertilizers were applied to ensure they were not limiting. Plant growth regulator was applied at GS31 (stem elongation) to reduce the risk of lodging. Herbicides were applied on 11 October 2022: Pontos 1 l ha^−1^ (BASF, Ludwigshafen, Germany), Wicket 3 l ha^−1^ (Syngenta, Basel, Switzerland), Backrow 0.2 l ha^−1^ (Interagro, Cambridge, UK). All field measurements were completed between 20 and 25 May 2023 at GS50 to 60 (inflorescence emergence) with variation depending on variety.

### Canopy biomechanical traits

Plant area index (PAI) (one‐sided green area per unit ground surface area) was measured with a ceptometer (Accupar LP‐80; Meter Group, Pullman, USA) on 22 May 2023, under a clear sky near solar noon; between 12:30 h and 15:30 h. Five measurements at mid‐canopy height and one above the canopy were taken per plot. Optical Chlorophyll content (SPAD‐502, Konica Minolta, Osaka, Japan) and flag LW were measured on the same day on five leaves per plot. Natural frequency (the rate at which a shoot sways) was calculated at the end of the experiment by a modified version of the assessment performed as part of lodging analysis (Berry *et al*., 2006). Two to three plants were selected per plot (nine per genotype) and removed from the field. A representative tiller was selected per plant and separated just above the root system. This was then anchored to a fixed point using a clamp against a white background. The tip of the ear was displaced 10 cm, and the movement was recorded using a Grasshopper 3 (Teledyne FLIR, Portland, OR, USA) camera, with a frame rate of 80 frames s^−1^. The time between the release and the completion of three oscillations was measured (*T, s*) based on video time stamps. This was repeated three times per plant, and natural frequency (*N*, s^−1^) was then calculated (Eq. 1)

(Eqn 1) $$N={\left(T/3\right)}^{-1}$$

Two additional plants per plot were harvested to measure tiller height (*H*, cm), leaf and total aboveground dry biomass (respectively LM and TM, g), total leaf area (LA, cm^−2^). From those, leaf mass per area (LMA, g cm^−2^) was calculated as (Eq. 2; Fig. S2).

(Eqn 2) $$\mathrm{LMA}=\frac{\mathrm{LM}}{\mathrm{LA}}$$

the height‐to‐mass ratio (HM, cm g^−1^) was calculated as (Eq. 3)

(Eqn 3) $$\mathrm{HM}=\frac{H}{\mathrm{TM}}$$

and the ratio of leaf‐to‐tiller mass (LTM, g g^−1^) was calculated as (Eq. 4)

(Eqn 4) $$\mathrm{LTM}=\frac{\mathrm{LM}}{\left(\mathrm{TM}-\mathrm{LM}\right)}$$

### Canopy light measurements and fleck analysis

Spectral measurements were recorded following the methods detailed in Hartikainen *et al*. (2018) and Durand & Robson (2023). Briefly, we used a CCD array spectroradiometer (Maya 2000 Pro; Ocean Insights, Dunedin, FL, USA) calibrated by the Finnish Radiation and Nuclear Safety Authority in April 2023 (STUK; Ylianttila *et al*., 2007), with a horizontally positioned cosine diffuser (D7‐H‐SMA; Bentham Instruments Ltd, Reading, UK) attached to the spectroradiometer by a fibre‐optic cable (FC‐UV400–2400 μm; Avantes, Leatherhead, UK). The small diffuser is a flat cylinder (4.2 × 1.8 cm), which can be placed inside dense canopies on a small tripod without affecting the canopy structure. We recorded four sets of 10 000 scans in each plot (*n* = 12; each including 1377 wavelengths in the range 280–898 nm) with an integration time of 10 ms using the ‘high‐speed acquisition’ routine of the spectrasuite software (v.2.0.162; Ocean insights, Dunedin, FL, USA). Above‐canopy measurements were recorded one to three times d^−1^. Two additional measurements with a darkening cap and a polycarbonate filter (blocking 280–400 nm radiation) were performed after each set to correct for baseline noise and stray light in the UV region, respectively. Processing of the spectra into irradiance values and implementation of the correction were performed with the ooacquire and photobiology packages (Aphalo, 2015; Aphalo & Ylianttila, 2022). Time series were recorded on 20, 21, and 24 May 2023, within 3 h of solar noon (at *c*. 13:30 h) in the absence of clouds, and at mid‐canopy height.

To detect and measure windfleck properties, we used the method detailed in Durand *et al*. (2021). In short, spectral irradiance was converted into PPFD, and the moment at which the rate of change between each time point of the time series crosses 0 indicates when PPFD switches from decreasing to increasing, or vice versa. From this, the start, peak, and end of a windfleck can be retrieved, as well as the associated PPFD. Those windflecks for which the increase in PPFD represented an increase between the peak and baseline of less than either 5% or 5 μmol m^−2^ s^−1^ were not considered, which eliminated the naturally noisy oscillations in irradiance. We measured windfleck duration (time difference between the start and end, s), intensity (PPFD difference between peak and baseline, μmol m^−2^ s^−1^), mean time between windflecks (time difference between the end of a windfleck and the start of the next, s), frequency (total number of windflecks recorded over the whole time series, s^−1^), and the intensity which is the integrated PPFD increase caused by the windfleck (subtracting a linearly interpolated PPFD between start and end to the total integrated PPFD during the windfleck, μmol m^−2^). See Durand *et al*. (2021) for illustrations of the windfleck traits measured.

Next to the spectroradiometer diffuser and a few centimetres above the canopy, a hot ball wind sensor with a data logger (Testo 440 data logger; Testo Inc., West Chester, PA, USA) was positioned horizontally to record wind speed every second at the same time as the spectroradiometer measurements. The wind sensor was positioned, such that it did not shade the diffuser.

### Motion analysis

On one plot per cultivar, additional video recordings were also performed simultaneously using a lightweight compact drone (320 g, Parrot ANAFI; Parrot Drones SAS, Paris, France) with a high‐resolution camera (21 million pixels, 30 frames s^−1^). To prevent the drone blades from producing significant wind that would move the wheat canopy below, the drone was attached to a 5‐m pole positioned to the side of the plot. Each video was stabilized with vegas pro v.21.0 (magix Software GmbH, Berlin, Germany) to compensate for translation, rotation, and scaling with an 18 × 12 grid and 20 points per grid cell.

Wind‐induced motion in the plot can be detected through changes in the colours of pixels (e.g. transition to darker or lighter RGB values). We can thus quantify the motion by calculating the difference in pixel value for each frame. To do this, we used the first frame of the video to manually define the vegetation plot area. The 2 × 1‐m plot area is converted into a 16 × 9 grid so that each grid cell was nearly square‐shaped, while also allowing division of the area into 23 zones (Fig. S3). To calculate the difference in pixel value, each frame ‘*n*’ of the video is compared to frame ‘*n* + 1’. For each pixel of each zone, we calculated the absolute difference in the red, green and blue. Then, the within‐zone variance of the absolute differences was summed for the red, green and blue to obtain a single value per frame that we defined as quantity of motion (QOM). Each red, green, and blue pixel can take any value between 0 and 1; thus, QOM is theoretically bound between 0 and 3 where 0 corresponds to no change in pixel value over the whole area, and 3 corresponds to a shift between pure white to pure black. Since differences in pixel values can also occur because of noise, three reference areas on the bare soil surrounding the plot, that did not move with the wind, were also used (Fig. S3). QOM within the three reference zones was averaged and subtracted to the QOM detected in each zone.

### Synchronizing the time series

**Fig. 1:**
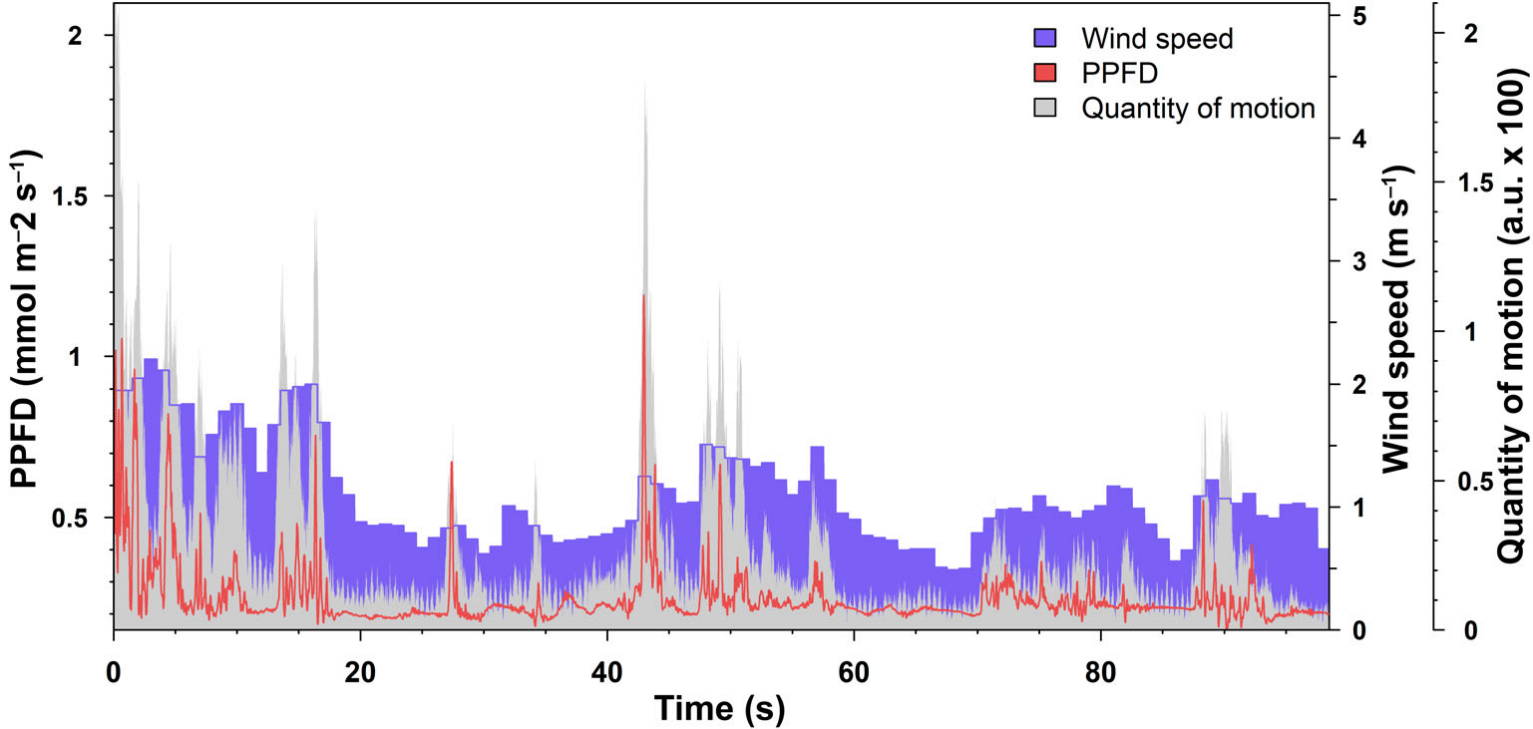
Example set of synchronized time series of photosynthetic photon flux density (PPFD) (in red), wind speed (in blue), and quantity of motion (QOM) (in grey) in the *Triticum aestivum* cultivar ‘Crusoe’, grown in the field in Nottingham, UK. PPFD is measured at a frequency of 100 s^−1^, QOM is interpolated from 30 s^−1^ to 100 s^−1^ during synchronization, but wind speed is extended without interpolation at 100 s^−1^ (hence the bar‐like shape of the time series).

Although all times‐series were recorded at the same time, small differences prevent automatic synchronization. To solve this, the PPFD time series was cross‐correlated to the QOM time series for each zone. We linearly interpolated QOM from 30 s^−1^ to 100 s^−1^ to mirror the measurement frequency of PPFD. We then retained a single zone which (1) was closest to the light diffuser where PPFD was recorded (accounting also for sun angle), and (2) had the largest absolute cross‐correlation value. To synchronize the wind speed time series, which had a slower measurement frequency (1 s^−1^), we extended the original data points, without interpolation, to a 100 s^−1^ frequency. We then computed the cross‐correlation between PPFD‐wind and QOM‐wind time series. This allowed us to determine the mean shift between time series, as the shift for which the product of the two cross‐correlations is largest. For this calculation, negative cross‐correlations between wind and QOM (implying that more wind produces less motion) were ignored. An example of the synchronized time series of PPFD, QOM, and wind is shown in Fig. 1.

### Statistical analyses

To test for significant differences among cultivars, we used a Type II ANOVA with the R (v.4.4.1; R Core Team, 2025) packages car (Fox & Weisberg, 2019), emmeans (Searle *et al*., 1980), and multcomp (Hothorn *et al*., 2008). Since the experiment was a split‐plot design with three blocks, mixed‐effects models were fitted with variety as a fixed effect and block as a random effect (*n* = 3 biological replicates per variety). Technical replicates (leaves, time series, and windflecks) were treated as technical observations and not as independent biological replicates. Statistical inference was therefore based on block‐level replication through the inclusion of block as a random effect. Mixed‐effects models naturally accommodate unbalanced sampling, and differences in the number of technical replicates among traits were accounted for without inflating the effective sample size. For traits derived from time‐series measurements, multiple discrete events were extracted within each cultivar × block combination and analysed without explicitly modelling time, thereby avoiding the use of individual time points as independent observations. For each trait, repeatability was estimated as the proportion of total variance explained by block effects, and broad‐sense heritability (*H* ^2^) was calculated following (Falconer & Mackay, 1996) from the variance among variety means and the residual variance within variety and block as:

(Eqn 5) $$H^{2}=\frac{\sigma_{V}^{2}}{\sigma_{V}^{2}+\frac{\sigma_{r}^{2}}{n}}$$

with $\sigma_{V}^{2}$ the variance among variety means, $\sigma_{r}^{2}$ the residual variance between variety means and variety × block means, and *n* the number of biological replicates (= 3 blocks). Scaling the residual variance by *n* accounts for replication, as environmental variance decreases when phenotypic values are averaged across biological replicates. In some cases, the block variance component was estimated as zero, indicating that block‐to‐block variation contributed negligibly to total variance for those traits. When measuring the natural frequency, three replicate measures were performed for each tiller/plant; thus, the plant was also defined as a random effect in the model. We checked for normality and homoscedasticity of the model residuals graphically, and performed *post hoc* pairwise contrast analyses to test for differences among cultivars. ANCOVA models were used to test for the effect of either wind speed or log‐transformed QOM on windfleck frequency for each cultivar after discretization. The significance of the slope per cultivar was performed with emmeans. Linear regressions were done using cultivar means to test for relationships between canopy traits and windfleck properties. We adjusted *P* values to control for the false discovery rate and significant differences were considered at *P* < 0.05 for all tests. We also performed a principal component analysis (PCA) using the trait data (Table 1), with the R package factominer (Lê *et al*., 2008). All data used in the PCA analyses were averaged per block and scaled. Data used in the analyses are available as Dataset S1.

**Table 1:** Summary of plant and canopy traits measured in 10 *Triticum aestivum* cultivars.

	Tiller height (cm)	Total fresh mass (g per plant)	Total leaf area (cm^2^)	Total leaf mass (g per plant)
Crusoe	77.5 ± 2.3 (0.03) bc	13.24 ± 2.15 (0.16) b	115.4 ± 18.9 (0.16) abc	2.64 ± 0.30 (0.12) bcd
*KWS Extase*	88.7 ± 4.4 (0.05) e	13.21 ± 1.50 (0.11) b	117.1 ± 9.9 (0.08) abc	2.63 ± 0.34 (0.13) bcd
*KWS Firefly*	76.6 ± 5.0 (0.07) bc	13.23 ± 2.34 (0.18) b	103.9 ± 13.8 (0.13) ab	2.39 ± 0.43 (0.18) ab
*KWS Kinetic*	83.1 ± 3.2 (0.04) d	13.50 ± 1.71 (0.13) b	134.4 ± 23.2 (0.17) c	2.94 ± 0.39 (0.13) cd
*Mayflower*	77.5 ± 4.1 (0.05) bc	12.02 ± 1.46 (0.12) ab	113.0 ± 6.4 (0.06) abc	2.64 ± 0.26 (0.10) bcd
*RGT Wilkinson*	74.8 ± 3.5 (0.05) ab	11.88 ± 2.01 (0.17) ab	108.8 ± 18.7 (0.17) abc	2.58 ± 0.46 (0.18) bc
*Skyfall*	78.5 ± 5.5 (0.07) c	11.60 ± 2.15 (0.19) ab	109.5 ± 23.2 (0.21) abc	2.35 ± 0.49 (0.21) ab
*SY Insitor*	75.0 ± 3.1 (0.04) ab	10.16 ± 0.89 (0.09) a	91.9 ± 12.7 (0.14) a	1.99 ± 0.15 (0.08) a
*Theodore*	76.3 ± 4.1 (0.05) bc	13.85 ± 1.79 (0.13) b	125.1 ± 25.7 (0.21) bc	3.08 ± 0.41 (0.13) d
*Zoom*	72.9 ± 2.6 (0.04) a	12.01 ± 1.00 (0.08) ab	107.9 ± 10.5 (0.10) abc	2.51 ± 0.10 (0.04) bc
Repeatability	0.09	0.00	0.05	0.05
Heritability	0.91	0.80	0.77	0.81

	Flag leaf width (mm)	Chl (SPAD index)	LMA (mg FW cm^−2^)	HM (cm g^−1^)
Crusoe	21.31 ± 1.08 (0.05) bc	52.0 ± 3.0 (0.06) c	23.1 ± 2.7 (0.07) abc	5.1 ± 0.5 (0.09) ab
*KWS Extase*	24.54 ± 1.10 (0.04) d	50.0 ± 3.0 (0.06) abc	22.4 ± 1.2 (0.05) ab	5.8 ± 0.6 (0.11) bc
*KWS Firefly*	21.88 ± 1.50 (0.07) c	52.2 ± 2.2 (0.04) c	22.9 ± 1.5 (0.07) ab	4.8 ± 0.7 (0.14) a
*KWS Kinetic*	23.42 ± 2.44 (0.10) d	49.9 ± 2.7 (0.05) abc	22.2 ± 3.0 (0.10) ab	5.1 ± 0.6 (0.12) ab
*Mayflower*	20.32 ± 2.18 (0.11) ab	47.8 ± 2.8 (0.06) a	23.4 ± 2.4 (0.10) ab	5.4 ± 0.4 (0.07) abc
*RGT Wilkinson*	23.31 ± 1.20 (0.05) d	50.3 ± 2.2 (0.04) bc	23.7 ± 1.8 (0.07) ab	5.1 ± 0.5 (0.10) ab
*Skyfall*	20.72 ± 1.60 (0.08) abc	49.9 ± 1.9 (0.04) abc	21.5 ± 1.1 (0.05) a	5.8 ± 0.9 (0.16) bc
*SY Insitor*	19.41 ± 2.32 (0.12) a	48.8 ± 1.7 (0.04) ab	21.9 ± 2.7 (0.06) ab	6.1 ± 0.6 (0.09) c
*Theodore*	21.56 ± 1.13 (0.05) bc	52.0 ± 4.1 (0.08) c	26.32 ± 1.11 (0.04) c	4.57 ± 0.55 (0.12) a
*Zoom*	21.50 ± 1.11 (0.05) bc	51.5 ± 2.0 (0.04) c	24.43 ± 1.59 (0.06) bc	5.02 ± 0.30 (0.06) ab
Repeatability	0.00	0.03	0.00	0.00
Heritability	0.84	0.85	0.85	0.87

	LTM (g g^−1^ FW)	LAN (cm^2^)	Plant area index (m^2^ m^−2^)	Natural frequency (s^−1^)
Crusoe	0.252 ± 0.022 (0.09) abc	26.06 ± 5.99 (0.23) ab	3.99 ± 0.97 (0.24) c	1.78 ± 0.22 (0.13) abc
*KWS Extase*	0.249 ± 0.021 (0.08) abc	27.52 ± 2.86 (0.10) ab	3.74 ± 1.49 (0.40) c	1.56 ± 0.19 (0.13) a
*KWS Firefly*	0.221 ± 0.009 (0.04) a	24.33 ± 2.74 (0.11) a	2.79 ± 0.86 (0.31) a	2.10 ± 0.26 (0.12) de
*KWS Kinetic*	0.279 ± 0.016 (0.06) bc	31.91 ± 7.40 (0.23) b	3.56 ± 0.88 (0.25) bc	1.80 ± 0.13 (0.07) abc
*Mayflower*	0.284 ± 0.034 (0.12) bc	23.37 ± 1.95 (0.08) a	3.56 ± 0.67 (0.19) bc	2.01 ± 0.18 (0.09) cd
*RGT Wilkinson*	0.280 ± 0.051 (0.18) bc	24.58 ± 3.03 (0.12) a	2.73 ± 0.39 (0.14) a	2.28 ± 0.10 (0.04) e
*Skyfall*	0.254 ± 0.029 (0.12) abc	23.61 ± 5.60 (0.24) a	2.87 ± 0.44 (0.15) ab	1.68 ± 0.18 (0.11) ab
*SY Insitor*	0.244 ± 0.007 (0.03) ab	22.99 ± 3.19 (0.14) a	3.59 ± 0.92 (0.26) bc	1.91 ± 0.26 (0.13) bcd
*Theodore*	0.288 ± 0.038 (0.13) c	24.41 ± 5.29 (0.22) a	2.98 ± 0.48 (0.16) ab	1.92 ± 0.17 (0.09) bcd
*Zoom*	0.267 ± 0.028 (0.10) bc	26.17 ± 2.98 (0.11) ab	3.35 ± 0.54 (0.16) abc	1.88 ± 0.20 (0.11) bcd
Repeatability	0.03	0.01	0.002	0.00
Heritability	0.77	0.80	0.70	0.87

Values are means ± SE (coefficient of variation). Letters represent statistically significant differences between groups tested by *post hoc* pairwise comparisons (*P* < 0.05). FW: fresh weight; HM, height‐to‐mass ratio; LAN: leaf area per leaf; LMA, leaf mass per area; LTM, leaf‐to‐tiller mass; PAI, plant area index. For all traits, *n* = 3 (three plots per variety in a randomized complete block design) with two to five technical replicates.

## Results

### Variability in motion produced by wind speed among cultivars

With increasing wind speed, motion increased exponentially in most cultivars (Fig. 3a). At high wind speed (> 1.5 m s^−1^), motion increased by at least 1.8 times (i.e. in KWS Firefly), and up to 5.5 times (i.e. in SY Insitor). Motion in KWS Firefly exhibited the lowest magnitude of change following an increase in wind speed (Fig. 3b), meaning its stem likely dampened motion. Overall Crusoe was the cultivar for which wind produced the least motion at both high and low (< 1.5 m s^−1^) wind speeds, suggesting a more stable canopy architecture that resists displacement. Contrary to this, wind consistently produced the most motion in Zoom and Mayflower when accounting for both low and high wind speeds. But when considering high wind speeds only, Skyfall exhibited the most motion, which was 22% higher than in Mayflower (second highest), and 4.6 times higher than that of Crusoe, indicating greater aerodynamic sensitivity.

In general, inconsistent cultivar rankings were seen for the QOM under low wind speeds vs higher wind speeds. Notably, SY Insitor and Skyfall had relatively low motion under low wind speed, but relatively higher (i.e. ranked higher out of cultivars) at high wind speed, implying that these cultivars are more susceptible to bending once aerodynamic forces exceed a threshold. By contrast, KWS Kinetic, KWS Firefly, and KWS Extase were ranked higher in terms of relative motion at low but not high wind speeds, suggesting a limit of additional movement once a certain deformation is reached. It should be noted that very little motion data could be collected for Theodore at wind speeds above 1.5 m s^−1^, which makes it difficult to interpret its behaviour under higher wind speed compared to other cultivars.

Overall, this analysis reveals distinct mechanical and aerodynamic strategies among cultivars: some maintain rigid, motion‐damping canopies (e.g. Crusoe and Firefly), whereas others (e.g. Skyfall and SY Insitor) exhibit greater flexibility that amplifies at higher wind speed.

### Trade‐offs in canopy structure at the cultivar level

**Fig. 2:**
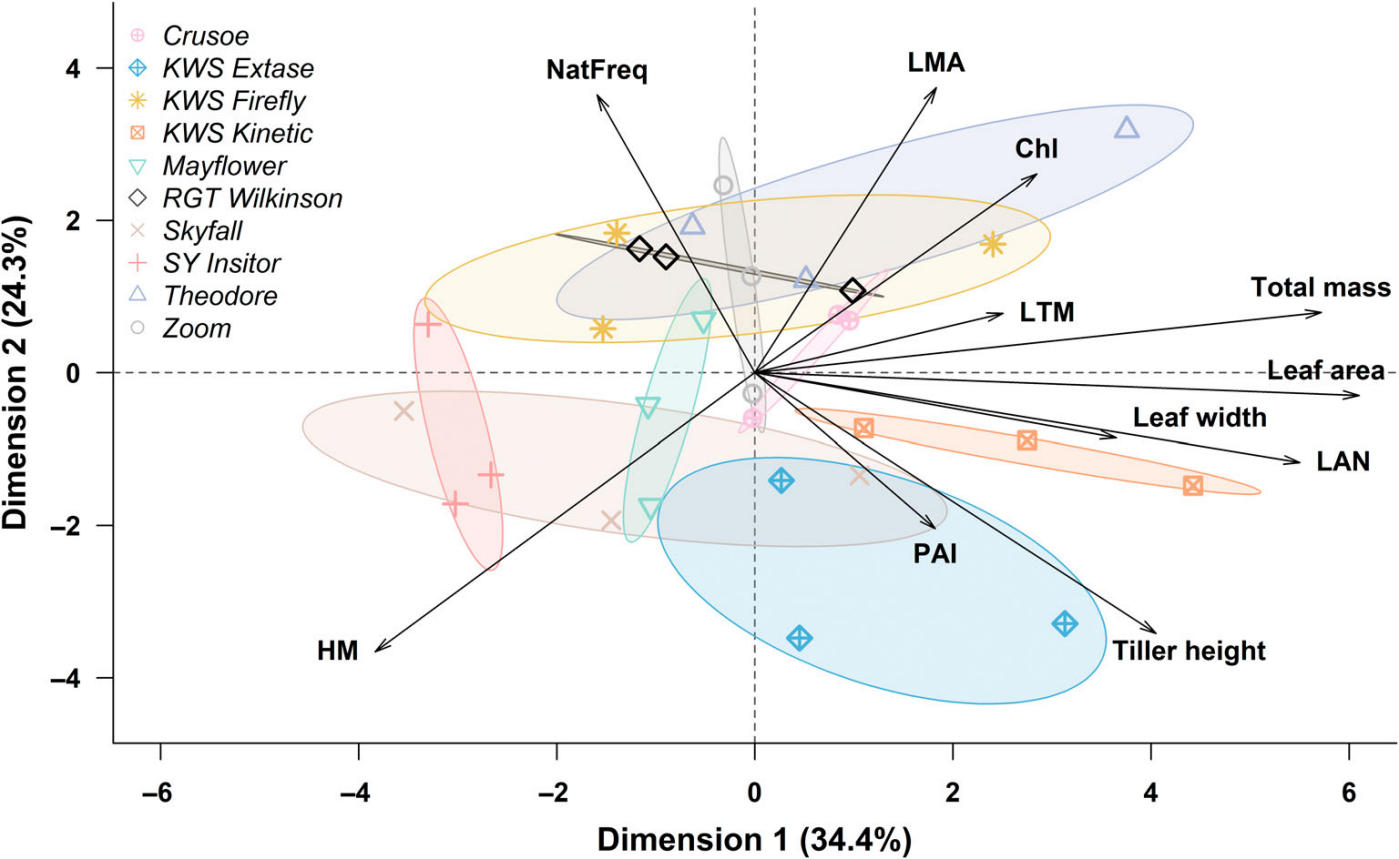
Principal component analysis of canopy structural traits of 10 *Triticum aestivum* cultivars grown in the field at Nottingham, UK (Crusoe, KWS Extase, KWS Firefly, KWS Kinetic, Mayflower, RGT Wilkinson, Skyfall, SY Insitor, Theodore, and Zoom). Traits include tiller height, total fresh mass, total leaf area, flag leaf width, Chl content, leaf mass per area (LMA), height‐to‐mass ratio (HM), total leaf per tiller mass (LTM), leaf area per leaf (LAN), plant area index (PAI), and natural frequency (NatFreq). Points are averages per block of two to three replicates.

The PCA revealed that the canopy structure among the 10 wheat cultivars was organized along two main axes (Fig. 2). First, there was a trade‐off whereby cultivars with stiffer and less flexible stems (i.e. high natural frequency) tended to form shorter and sparser canopies with lower tiller height and PAI. For example, RGT Wilkinson and KWS Firefly had a 46% and 35% higher natural frequency, respectively, than KWS Extase (*P* < 0.002 and *P* < 0.02; Table 1). Reciprocally, KWS Extase had a 37% and 34% higher PAI than RGT Wilkinson and KWS Firefly, respectively (*P* < 0.001 and *P* < 0.02). KWS Extase also had a 19% and 16% higher tiller height than RGT Wilkinson and KWS Firefly, respectively (in both cases *P* < 0.0002). Overall, the PCA explained 71.3% of the total variance in the first three axes. The first axis generally represented canopy size and biomass production as both total LA and total fresh mass contributed most to the axis. LMA, HM, and natural frequency contributed most to the second axis, while LTM contributed most to the third axis. Together, these patterns indicate that mechanical stiffness and canopy density are inversely related, suggesting that cultivars investing in stiffer, load‐bearing stems may do so at the expense of leaf deployment and overall canopy expansion.

The second trade‐off was between cultivars with a large HM, but low LMA and Chl content (Fig. 2). For example, SY Insitor had the highest HM, 33% higher than Theodore, which had the lowest (*P* < 0.0004; Table 1). Reciprocally, Theodore had an LMA and Chl content 15% and 7% higher, respectively, than in SY Insitor (*P* < 0.008 and *P* < 0.007). Skyfall and Mayflower also tended to have high HM but low LMA and Chl content (Fig. 2). By contrast, both Zoom and Crusoe were positioned intermediately along the two trade‐off axes, but KWS Kinetic was unique in showing the highest total LA, and the second highest flag LW, tiller height, and total fresh mass (Table 1). These structural contrasts reveal that cultivars emphasizing structural stability (high HM, high frequency) tend to limit leaf development and Chl concentration, while those with greater canopy investment may trade off mechanical rigidity for increased light‐capture potential.

### Distinct windfleck properties between wheat cultivars resulting from wind‐induced motion

After examining how wind produces motion in wheat, our next step was to determine how the light environment in a wheat canopy changes as plants move. Although data with a higher QOM is noticeably noisier due to less data availability, we nevertheless identified clear cultivar‐specific effects of motion on the duration and intensity of windflecks, and thus on the integrated PPFD increase due to the windfleck (Fig. 4a). Among them, Skyfall, Zoom, KWS Firefly, and SY Insitor consistently and significantly showed the highest integrated PPFD increase (compared to all other cultivars: *P* < 0.03, except between SY Insitor and KWS Extase: *P* = 0.13; Table 2), while RGT Wilkinson consistently showed the lowest integrated PPFD increase (compared to all other cultivars: *P* < 0.03, except with Mayflower and Crusoe: *P* > 0.29; Table 2). These patterns suggest that more dynamically deforming canopies (e.g. Skyfall, Zoom) produce sharper and more frequent flecks of light that enhance PPFD within the canopy.

**Table 2:** Summary of windfleck properties measured in 10 *Triticum aestivum* cultivars.

	Windfleck intensity (μmol m^−2^ s^−1^)	Windfleck duration (s)	Integrated PPFD increase (μmol m^−2^)	Windfleck frequency (100 s^−1^)	Windfleck number
Crusoe	98.1 ± 6.3 (1.34) a	0.181 ± 0.005 (0.53) bcd	11.97 ± 1.05 (1.83) ab	122.8	436
*KWS Extase*	154.1 ± 8.7 (1.13) b	0.193 ± 0.006 (0.65) cd	22.07 ± 2.08 (1.89) cd	130.3	405
*KWS Firefly*	202.8 ± 11.8 (1.24) c	0.233 ± 0.005 (0.48) e	28.02 ± 1.98 (1.51) e	146.3	454
*KWS Kinetic*	108.2 ± 8.2 (1.28) a	0.222 ± 0.008 (0.57) e	16.15 ± 1.85 (1.93) bc	84.8	285
*Mayflower*	108.3 ± 5.3 (1.22) a	0.170 ± 0.004 (0.53) ab	11.85 ± 0.83 (1.75) ab	176.6	628
*RGT Wilkinson*	89.4 ± 7.7 (1.56) a	0.158 ± 0.004 (0.47) a	9.04 ± 0.94 (1.89) a	114.9	328
*Skyfall*	281.4 ± 10.1 (1.00) d	0.222 ± 0.005 (0.60) e	36.98 ± 1.84 (1.39) f	218.1	774
*SY Insitor*	221.5 ± 21.7 (1.32) c	0.174 ± 0.007 (0.55) abc	27.49 ± 3.54 (1.74) de	145.1	182
*Theodore*	139.4 ± 7.3 (1.26) b	0.196 ± 0.005 (0.59) d	19.13 ± 1.54 (1.94) c	158.2	578
*Zoom*	311.9 ± 14.0 (0.81) e	0.161 ± 0.004 (0.47) a	31.27 ± 2.05 (1.18) e	134.8	323

Values are means ± SE (coefficient of variation). Letters represent statistically significant differences between cultivars tested by *post hoc* pairwise comparisons (*P* < 0.05). For all traits, *n* = 3 (three plots per variety in a randomized complete block design) with additional technical replicates (windflecks recorded in each time series). [Correction added on 27 February 2026: the units in the ‘Windfleck frequency’ column have been updated.]

Windfleck duration was largely unaffected by changes in motion (Fig. 4b). KWS Firefly, KWS Kinetic, and Skyfall had overall the longest windflecks (compared to all other cultivars: *P* < 0.002; Table 2), lasting on average 41% longer than in RGT Wilkinson and Zoom, which had the shortest windflecks. By contrast, windfleck intensity increased strongly with motion (Fig. 4c). Zoom consistently had the highest windfleck intensity (compared to all other cultivars: *P* < 0.03; Table 2), but its shortest duration (not significantly different from RGT Wilkinson, Mayflower, and SY Insitor; *P* > 0.23) still resulted in the second highest integrated PPFD increase. By contrast, Skyfall and KWS Firefly had both high intensity (above all other cultivars except Zoom: *P* < 0.0008) and long duration (above all other cultivars except KWS Kinetic: *P* < 0.002), producing the greatest integrated PPFD increase (above all other cultivars except Zoom and SY Insitor: *P* < 0.03). RGT Wilkinson had consistently the shortest and least intense windflecks, even under strong motion (Table 2). In Theodore, windfleck intensity was low at modest quantities of motion but increased sharply with stronger motion (Fig. 4c). Overall, differences in canopy architecture and flexibility determine whether motion translates into brief, intense light bursts or into longer, lower‐intensity events. Cultivars with both flexible stems and open canopy structure (e.g. Skyfall and Firefly) are particularly effective at converting motion into transient light enrichment. Differences in windfleck duration between cultivars were largely independent of changes in the QOM, whereas windfleck intensity was the major determinant for cultivar‐specific differences in the increase in the integrated amount of PPFD due to windflecks.

**Fig. 3:**
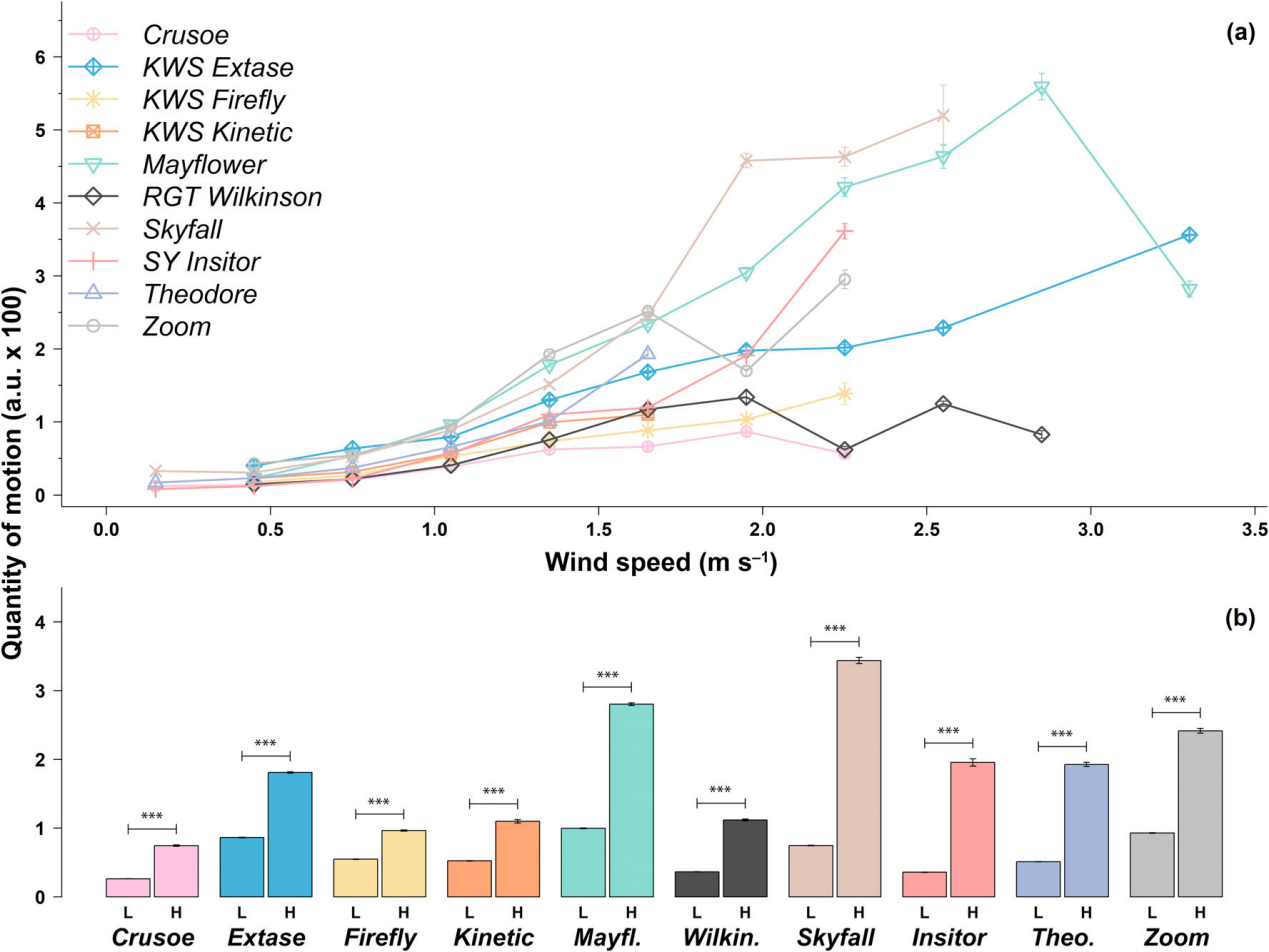
Quantity of motion produced by different wind speed in 10 *Triticum aestivum* cultivars grown in the field at Nottingham, UK. (a) For each cultivar, wind speeds were grouped in classes of 0.3 m s^−1^. (b) Wind speeds were bucketed in two classes: low (L, < 1.5 m s^−1^) and high (H, > 1. 5 m s^−1^). Values are means ± SE. ***, *P* < 0.0001. *n* = 3 (three plots per variety in a randomized complete block design).

Windfleck frequency increased from low to high wind speed and from modest to strong motion. Average windfleck frequency rose from 0.72 windfleck s^−1^ at wind speeds of 0.3–0.6 m s^−1^ to 2.49 windfleck s^−1^ at 1.8–2.1 m s^−1^: an almost 8‐fold increase (Fig. 5a). However, cultivar responses differed. In KWS Kinetic and KWS Firefly, windfleck frequency remained nearly constant beyond 1 m s^−1^ (*P* = 0.68 and *P* = 0.01 respectively), resulting in some of the lowest windfleck frequencies at high wind speed. Nevertheless, even if windfleck frequency showed almost no increase with wind speed in these cultivars, it did increase with motion (*P* < 0.003; Fig. 5b), indicating that limited canopy movement in these cultivars (Fig. 3a) still produced detectable light fluctuations once motion occurred. This contrasts with KWS Extase and RGT Wilkinson, where windfleck frequency increases relatively little with wind (although significant *P* < 0.009) or motion (*P* < 0.01; Fig. 5b). Conversely, SY Insitor and Mayflower had the highest windfleck frequency at high wind speed, followed by Skyfall and Theodore (in all cases *P* < 0.0001; Fig. 5a). This ranking was generally conserved when assessing windfleck frequency by motion, with SY Insitor, Skyfall, and Theodore consistently producing the most frequent windflecks under strong motion (*P* < 0.0001). These patterns reveal that the efficiency of light modulation depends not only on the canopy's mechanical response to wind but also on how its geometry translates motion into intermittent light exposure.

**Fig. 4:**
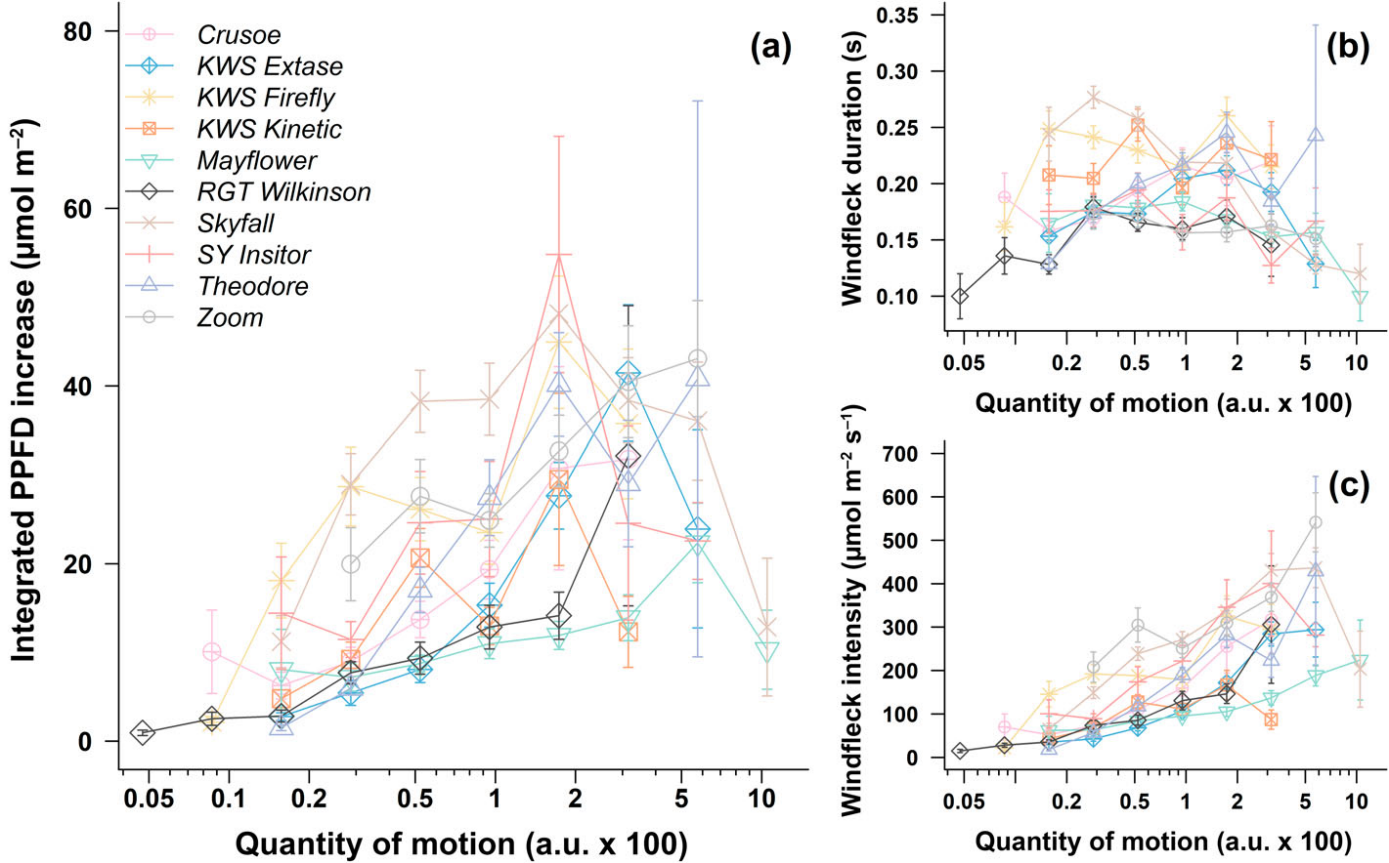
Properties of windflecks at different quantity of motion (QOM) in 10 *Triticum aestivum* cultivars grown in the field at Nottingham, UK. Windfleck properties include (a) the integrated photosynthetic photon flux density (PPFD) increase during the windfleck, (b) windfleck duration, and (c) windfleck amplitude. For each cultivar, QOM was grouped in 15 exponentially growing classes from e^−11^ (*c*. 0.0000167) to e^−2^ (*c*. 0.135). This was performed to equilibrate the number of observations in each class and get more accurate frequency estimation in each class. Classes for which one or fewer windflecks were found are not shown. Note the logarithmic scale on the *x*‐axis. Values are means ± SE.

**Fig. 5:**
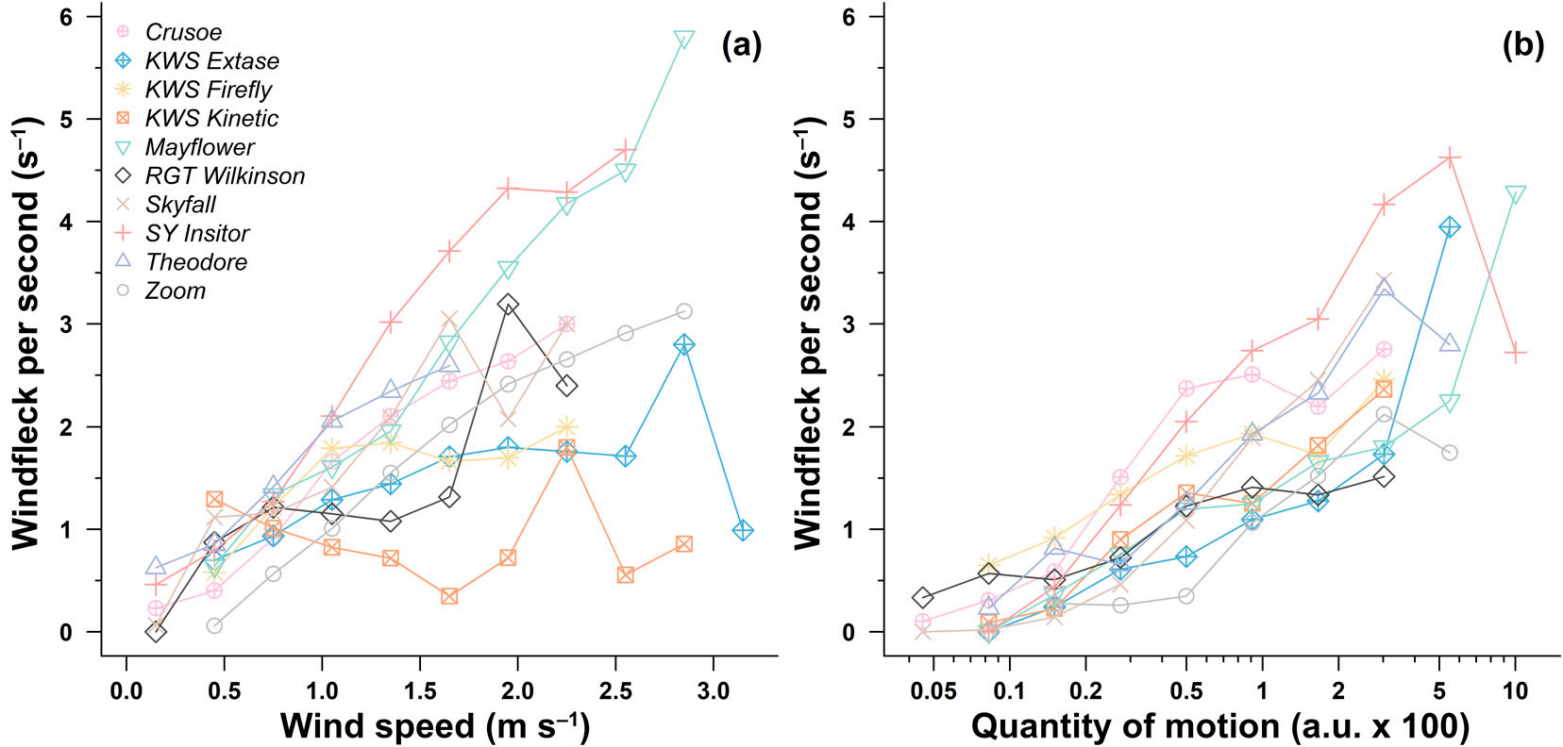
Frequency of windfleck occurrence at different wind speeds in 10 *Triticum aestivum* cultivars grown in the field at Nottingham, UK. (a) For each cultivar, wind speed was grouped in classes of 0.3 m s^−1^. (b) For each cultivar, quantity of motion (QOM) was grouped in 15 exponentially growing classes from e^−11^ (*c*. 0.0000167) to e^−2^ (*c*. 0.135). This was performed to equilibrate the number of observations in each class and get more accurate frequency estimation in each class. Note the logarithmic scale on the *x*‐axis. Frequencies were calculated as the number of windfleck over the time in each wind speed and QOM class, resulting in a single value per class.

**Fig. 6:**
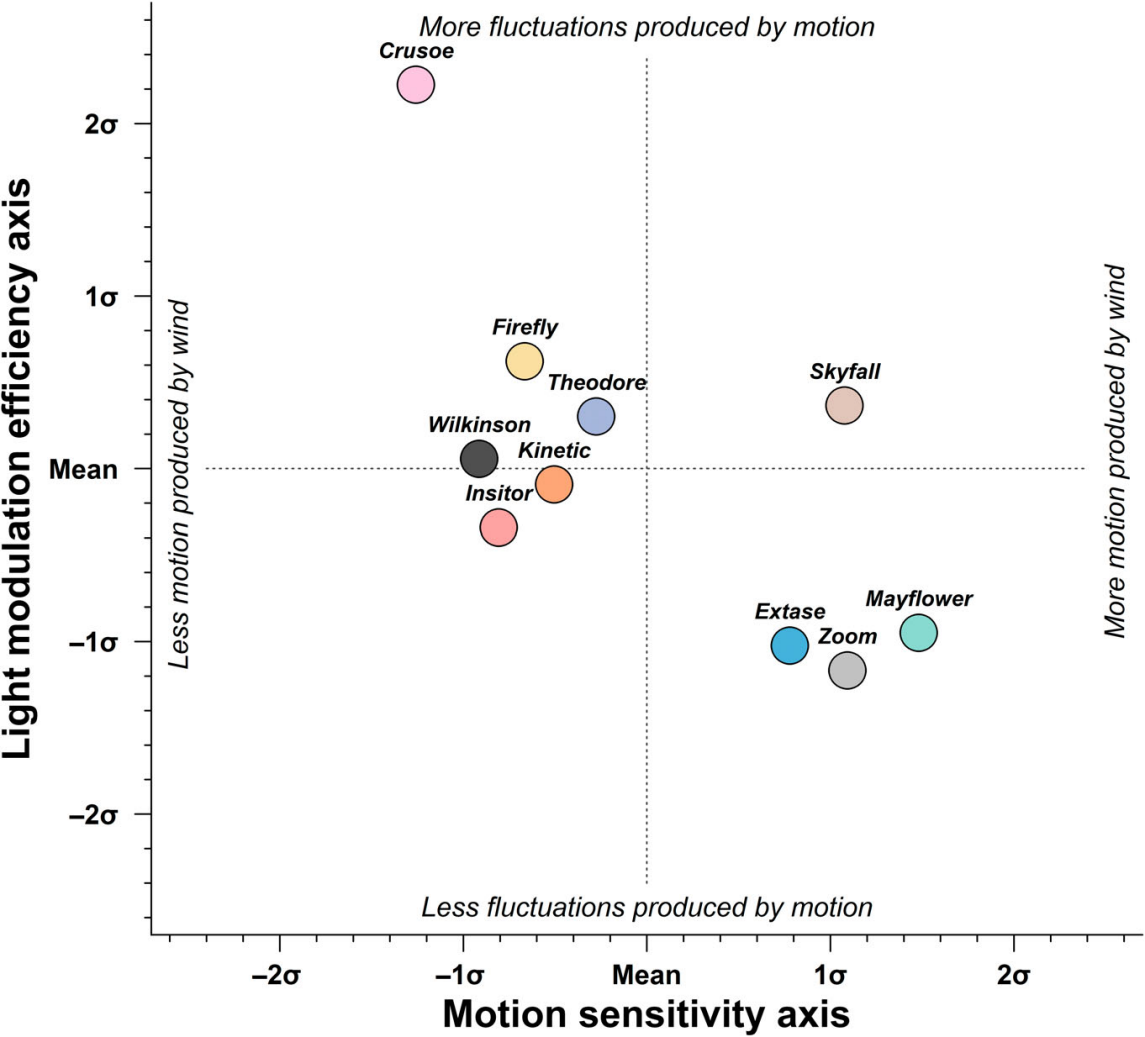
Representation of 10 *Triticum aestivum* cultivars grown in the field based on their capacity to produce motion under wind, and to produce windflecks when moving. The *x*‐axis is the motion sensitivity axis and is calculated for each cultivar over the whole recorded data as the ratio of mean motion to mean wind speed. The *y*‐axis represents the light modulation efficiency axis and is calculated as the ratio of windfleck frequency to mean motion. Values were centred and scaled so that a value of 0 represents the average of all cultivars, and a value of 1 represents a value of one SD (σ) above the mean.

Although Figs 4 and 5 allow us to describe how many windflecks are produced at high and low wind speed or motion, most of the recorded data were performed at wind speeds between 0.5 and 1.5 m s^−1^, and for motion between 0.001 and 0.006 (Fig. S4). Therefore, we plotted cultivars along two axes representing (1) ‘motion sensitivity’ of the cultivar, calculated as the ratio of average motion to average wind speed, and (2) ‘light modulation efficiency’, calculated as the ratio of windfleck frequency to average motion (Fig. 6). While the two axes were weakly correlated (*P* > 0.03), two main cultivar groups emerged. KWS Firefly, KWS Kinetic, Theodore, RGT Wilkinson, and SY Insitor were closest to the overall average on both axes, showing intermediate efficiency at converting motion into fluctuating light. By contrast, wind produces strong motion in Mayflower, KWS Extase, and Zoom relative to other cultivars. Yet, such motion was less efficient at producing light fluctuations in these than in the other cultivars. By contrast, motion in Skyfall was sensitive to wind, and was efficiently converted into windflecks, while Crusoe stood out as the cultivar for which wind produced the least motion, yet when motion occurred, it was the most efficient at producing windflecks (Fig. S1). This classification highlights how canopy mechanics and architecture jointly regulate the dynamic light environment. Cultivars like Skyfall, which are both mobile and efficient at converting motion into fluctuations, may experience greater within‐canopy light heterogeneity under natural wind conditions.

### Correlations between canopy traits and windfleck properties

**Fig. 7:**
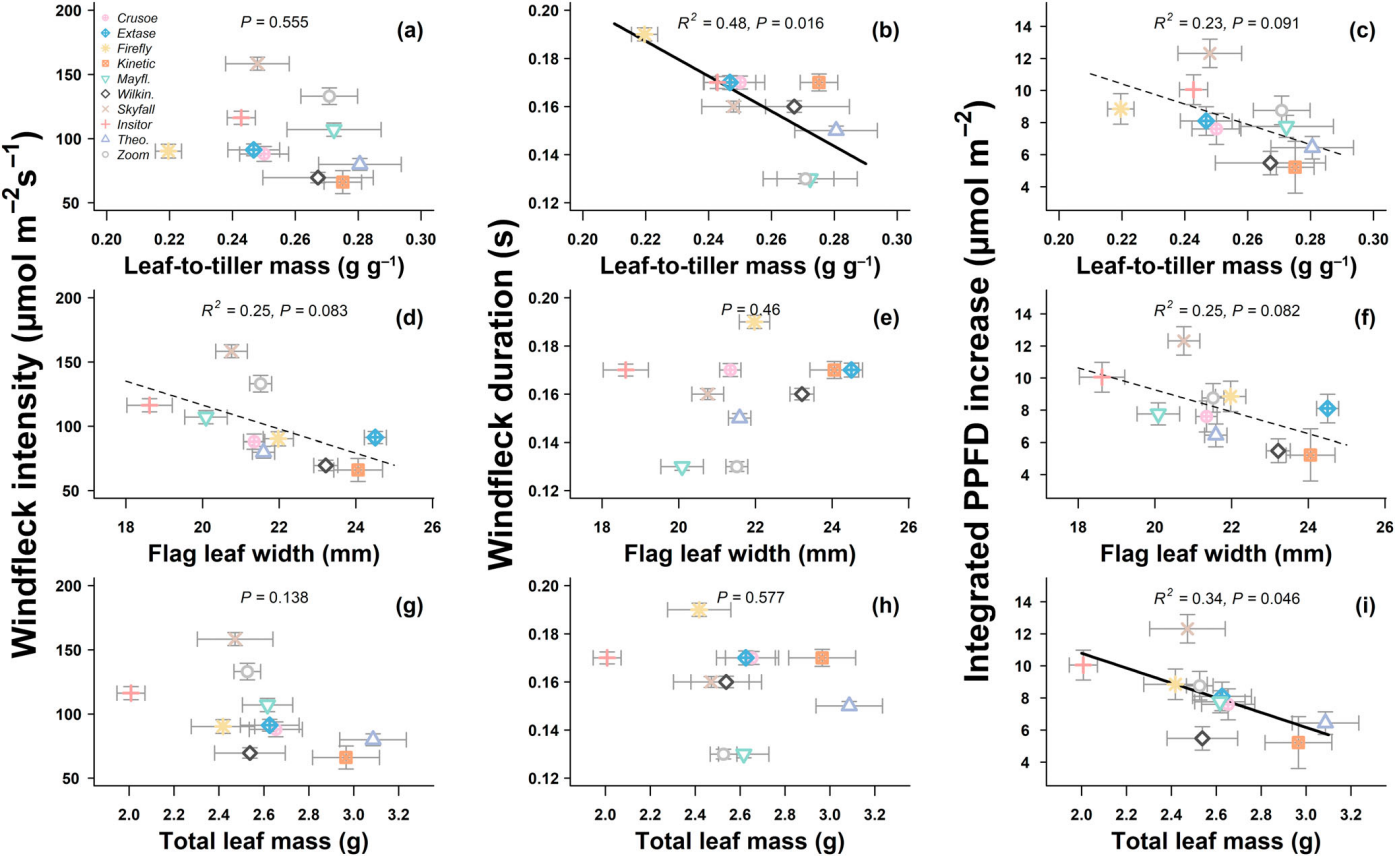
Correlations between windfleck properties and canopy structure in 10 *Triticum aestivum* cultivars grown in the field at Nottingham, UK. Windfleck intensity (a–g), windfleck duration (b–h), and integrated photosynthetic photon flux density (PPFD) increase (c–i) are shown in relation to leaf‐to‐tiller mass (LRM, a–c), flag leaf width (d–f), and total leaf mass (g–i). *P* value and *R* ^2^ of the linear regression are shown. Solid and dashed lines represent respectively significant (*P* < 0.05) and near‐significant (*P* < 0.1) trends. Values are median ± SE after the block effect was accounted for by adding the residuals of a linear model with the block as main effect to the global mean.

Windfleck properties cannot be directly paired with canopy structural traits at the individual measurement level; thus, we had to use cultivar means to test for correlations. This approach inevitably reduces statistical power, as each cultivar is considered as a single replicate even though it is informed by many observations. Most correlations were generally weak or near‐significant (i.e. 0.05 < *P* < 0.1), which likely reflects both this limitation and biological variability among cultivars. The supplementary correlations shown in Fig. S5 were not statistically significant, further confirming the modest strength of these relationships. The integrated PPFD increase due to windflecks was significantly negatively correlated with leaf mass (*P* = 0.04; *R* ^2^ = 0.34), and nearly significantly negatively correlated with both LTM (*P* = 0.09; *R* ^2^ = 0.23) and flag LW (*P* = 0.08; *R* ^2^ = 0.25; Fig. 7). These patterns suggest that canopies composed of lighter, narrower leaves tend to produce more dynamic light fluctuations, whereas those with larger or heavier leaves dampen such variability. Further examining individual components, windfleck duration was significantly negatively correlated with LTM (*P* = 0.02; *R* ^2^ = 0.48; Fig. 7b), but not with either flag LW (*P* = 0.46) or leaf mass (*P* = 0.58). On the contrary, windfleck intensity was not correlated with LTM (*P* = 0.55), but a negative correlation with flag LW was nearly significant (*P* = 0.08; *R* ^2^ = 0.25; Fig. 7d), unlike with leaf mass (*P* = 0.14). Overall, traits related to the amount of leaf material tended to reduce windfleck intensity, while windfleck duration tended to be diminished in cultivars with larger LTM.

## Discussion

### Investigating light fluctuations *in situ* via remote sensing of motion

In this study, we aimed to use a novel method to assess how the motion of wheat cultivars changes under different wind speeds and the corresponding impact of light fluctuations within the canopy. The method we used, based on quantifying the difference in pixel value between frames (also known as frame differencing) as an indication of plant motion, allowed us to infer how wind produces fluctuations in light within the canopy via creating movement. To our knowledge, this is the first time this method has been applied to investigate how the *in canopy* light environment is shaped by plant movement, which allowed us to answer novel research questions. Our results show that (1) the same wind speed produces a diversity of motion in wheat cultivars, (2) increased motion is related to increased windfleck frequency and intensity, but not duration, and (3) canopy structure does determine windfleck properties, even though several of the biomechanical traits we measured did not show the significant correlations with windfleck properties that we had initially anticipated.

These findings are significant as they provide the foundations to identify the combination of traits that may be associated with a given *in canopy* light environment. One striking finding of our study is the considerable differences in motion between wheat cultivars (Fig. 3). Because the cultivars used in this study are all high yielding and commercially grown, one may expect further variability in regionally adapted, older, or conservation cultivars of wheat. There is thus clear potential to identify beneficial traits facilitating characteristics of wind movement which stimulate canopy photosynthesis using existing germplasm. Although our work focused on the inflorescence emergence stage, it is very likely that the influence of wind on canopy movement will also be dependent on phenology, and more broadly on geographical and topological location (Burgess *et al*., 2019). Therefore, developing tools to stimulate more efficient canopy movement will necessitate a broader understanding of the factors determining plant motion.

The principle of frame differencing has been used previously to examine crop failure under high wind (Susko *et al*., 2019). Although previously only the mean of the red channel was recorded, we used the variance of the full RGB space which, from testing, we found to be less sensitive than the mean to video artefacts and automatic exposure correction by the camera. Artefacts were more common using .jpg files than .png, and depend on the subject recorded; thus, the reference zone could not completely subtract all artefacts from the QOM. Nevertheless, frame differencing is a method used in diverse applications, such as to quantify fidgety movement in infants (Adde *et al*., 2009). Through remote and nondestructive quantification of motion, this opens the possibility to investigate the impact of leaf flutter on radiative and carbon balance (e.g. in poplar trees; Roden & Pearcy, 1993), stem displacement, and strain to understand determinisms of thigmomorphogenesis in trees (Dlouhá *et al*., 2024), and could be coupled with thermal cameras to map out how turbulent flow and plant movement affect the canopy boundary layer and leaf temperatures.

### Are plant biomechanical characteristics governing the properties of light fluctuations?

Our examination of canopy structure and biomechanical properties revealed two trade‐offs whereby larger HM was associated with lower LMA and Chl content, while on the contrary, higher natural frequency was associated with lower PAI and tiller height. The first trade‐off reflects a strategy linked with competition for light, with some cultivars prioritizing light capture through reduced structural density rather than higher Chl content. Such traits are also often associated with fast‐growing plants and shorter leaf lifespans (Wright *et al*., 2004). This can translate into higher yield, as Skyfall was found to be among the highest yielding cultivars in comparison with 19 others (Camenzind & Yu, 2023).

While we expected that lighter cultivars (i.e. less dense plant material) may lead to increased motion in the wind, our analysis did not find strong evidence in favour of this hypothesis. A possible explanation may be due to a dampening effect on individual plant motion. The dense arrangement of tillers within a plot results in each tiller's motion being not only determined by its biomechanical properties, but also by the presence of neighbouring tillers (Doaré *et al*., 2004). The same effect led us to systematically exclude plants closest to the plot border because they exhibit the most motion in the wind (Fig. S3).

The second trade‐off separates cultivars such as KWS Extase, which displayed tall stems and denser canopies (i.e. high PAI), against cultivars such as KWS Firefly and RGT Wilkinson, which instead were able to vibrate their stems faster (i.e. high natural frequency). However, natural frequency and PAI did not directly predict windfleck properties. Instead, windfleck properties seemed most correlated to traits relating to aerodynamic drag. Windfleck intensity and duration were negatively correlated with both flag LW and LTM (Fig. 7). A high LTM corresponds to a tiller with relatively more leaf biomass compared to its stem, thus creating a more blunt shape increasing drag (Mason & Lee, 1994). In agreement with this hypothesis, the integrated PPFD increase due to the windfleck was also associated with lower leaf mass and LW. Apart from the effect on drag, lower leaf mass and area may contribute to a larger integrated increase in PPFD by producing larger gaps in the canopy, which reduces penumbral effects (Smith *et al*., 1989).

Perhaps surprisingly, those cultivars which moved more easily in the wind were not always the ones that exhibited the largest windfleck frequency (Fig. 6). Indeed, the ability to move in the wind does not seem to systematically result in a better ability to alter the light environment when moving. This reflects previous studies on streamlining in giant reed (*Arundo donax*), whereby reorienting leaf material even under low wind speeds (< 1 m s^−1^) is predicted to be an important adaptation for withstanding high wind loads and preventing mechanical damage (Speck, 2003).

In this study, motion recorded by the camera corresponds to movement of leaves and tillers within the canopy. Yet, this method is not able to differentiate between different kinds of movement by the plant (e.g. stem rotation, and lateral and vertical leaf wobble, see fig. 1 in Burgess *et al*., 2019). Specific architecture, biomechanical properties, and the positioning of neighbouring plants may affect the type of motion experienced, in turn affecting the efficiency at which motion is converted into fluctuating light within the canopy. It should also be noted that each movement type may not be captured equally efficiently by our method, which could bias our cultivar comparison. In this study, we measured during the same time of day, with the same camera angle and distance to the plants to ensure the same lighting and surface of ground captured, increasing comparability between varieties. While we have not specifically assessed their impact, these factors need consideration in future studies using this method to guarantee comparability between studies. Ideally, movement should be captured under overcast conditions (removing the effect of sun angle), when leaves are neither shaded nor brightly sunlit, allowing better capture of movement deep in the canopy. Yet, this would have prevented recording windflecks at the same time. In theory, differences in architecture (e.g. leaf angles) could not only affect how plants move in the wind, but also how their movement is captured by this method. While this is true of most new methods that cannot be compared to a reference, it is important to bear in mind when interpreting our results. Nevertheless, whether the ability to produce movement in the wind is truly decoupled from the ability to produce light fluctuations when moving will require further determination of the governing factors of each process. Still, our results draw attention to a potential to modulate the canopy light environment by breeding cultivars efficiently producing fluctuating light such as Crusoe, with cultivars more sensitive to wind such as Mayflower or Skyfall.

While we focused on wheat with erect leaves, further studies are required to study the broader implications of movement covering a variety of crop species and architectures. Wheat varieties with drooping leaves will likely produce different motion in the wind, due to leaves partly resting on other canopy elements, affecting the light environment. It is likely that other cereals may achieve a similar range of motion, whereas by contrast, broadleaf species may exhibit significantly different motion as a result of contrasting structural traits (Burgess *et al*., 2019). The response of a vegetative organ to wind depends upon its length, surface area, tensile strength, and mass, and the connectivity to other organs. For stem structures, low strength and a large mass can lead to breakage, whereas for leaves, mass and surface area will influence movement, particularly fluttering or twisting. The range of motion will also depend upon water status (Derzaph & Hamilton, 2013; Gonzalez‐Rodriguez *et al*., 2016). In addition to structural traits, management practices such as planting density will influence the capacity for any individual plant to move as a result of interactions with their neighbours (Doaré *et al*., 2004). Therefore, the specific biomechanical properties which determine light fluctuations will differ for each crop species and may also present some degree of year‐to‐year difference depending on the weather.

### Which patterns of motion and windflecks optimize photosynthesis?

Physiological responses to fluctuating light can generally be separated into slow vs fast. Slow responses involve processes, such as stomatal opening and closure (Allen & Pearcy, 2000; McAusland *et al*., 2016), Rubisco activation and deactivation (Taylor & Long, 2017; Taylor *et al*., 2022), and photosynthetic acclimation (Retkute *et al*., 2015; Townsend *et al*., 2018). Such processes can last from a few minutes to more than an hour; however, 95% of the windflecks we measured lasted between 0.05 and 0.44 s. Nevertheless, such fluctuations are an important feature of crop canopies (Durand & Robson, 2023), especially since penumbral effects and the occurrence of patches of sunlight due to gaps in the canopy (i.e. ‘sunflecks’) are much less common than in tall mixed forests (Smith *et al*., 1989; Smith & Berry, 2013).

Recent research has found evidence that faster recovery from photoprotection (typically lasting 1–2 min) leads to a 15% increase in biomass production in *Nicotiana tabacum* and a 33% increase in seed yield in *Glycine max* (Kromdijk *et al*., 2016; De Souza *et al*., 2022). Windflecks tend to be clustered together as a result of wind eddies (Fig. 1). As such, there are appreciable periods of time at which the canopy is still, under which faster recovery of photoprotection in the shaded parts could benefit photosynthesis. Cultivars which are more sensitive to motion in the wind could also more easily rearrange the position of their stems and leaves, allowing different parts of the canopy to be shaded. Rapid light fluctuations are also known to maintain stomata open (Zeiger *et al*., 1985) and Rubisco active (Tanaka *et al*., 2019), thus enabling windflecks to enhance carbon gain even when they are infrequent.

Fast responses to fluctuating light involve a three‐step process, whereby the light‐activated RuBP regeneration and residual metabolite pools lead to CO_2_ assimilation less than half a second after a light increase (Sassenrath‐Cole & Pearcy, 1992). Post‐windfleck, residual photosynthetic metabolite pools are also responsible for sustained CO_2_ assimilation up to several seconds (Laisk *et al*., 1984), which contributes a significant proportion of the total carbon gained in short fluctuations (Pons & Pearcy, 1992). In light fluctuations longer than 10 s, a burst of residual photorespiratory metabolites can offset overall the CO_2_ assimilated (Vines *et al*., 1983; Pearcy, 1990). More broadly, repeated windflecks on leaves may also impact leaf temperature, and thus photorespiration and transpiration rates (Durand *et al*., 2024). While these processes have been described more than 30 years ago, only recently – with our assessment of the intensity, duration, and frequency of windflecks – can we begin to assess how much they contribute to crop photosynthesis. Furthermore, evidence from modelling has shown that frequent windflecks facilitated by easier motion in the wind lead to light penetrating deeper in the canopy. The resulting light distribution within the canopy is thus more vertically homogenous, increasing overall canopy photosynthesis (Burgess *et al*., 2016, 2019).

Whether traits conferring a more static or dynamic canopy would be more desirable is difficult to judge, as either adjustment could benefit photosynthesis if part of a broader strategy. A static canopy produced by breeding for mechanical stiffness, installation of windbreaks, and located in less windy locations (e.g. valleys) would benefit more from fast stomatal responses and NPQ relaxation because when movement does happen, the newly sunlit and shaded leaves would reach their new regime faster. However, less fluid canopies are unlikely to be considered desirable (Dupont *et al*., 2025). On the contrary, more dynamic canopies would benefit most from faster RuBP regeneration – for *example* from enhanced ATP and NADPH production (Driever *et al*., 2017), or overexpression of SBPase (Driever *et al*., 2017).

### Conclusion

We found that even in relatively similar high‐yielding wheat cultivars, there were extensive differences in the QOM produced by the same wind speed. Moreover, cultivars were not equal in their ability to produce windflecks when moving. Finally, we found that windfleck properties were principally linked with traits related to the aerodynamic drag of the plant. Altogether, our results show the potential to optimize the dynamic canopy light environment for photosynthesis in wheat using plant biomechanics. Before such an objective can be accessed, future research will need to consider the kind of fluctuating light most desirable to optimize photosynthesis, and which plant movement produces the desired fluctuating light. Only by embracing how leaves move to modulate light can we design crops that harness the full potential of the complex dance between wind and light to thrive in a turbulent world.

## Competing interests

None declared.

## Author contributions

MD, AJG , TMR, and EHM developed the experimental design. MD, AJG and JAG collected the data. MD analysed the data. MD and AJG wrote the initial draft of the manuscript. MD, AJG, JAG, EHM, and TMR contributed to the result interpretation and edition of the final manuscript.

## Disclaimer

The New Phytologist Foundation remains neutral with regard to jurisdictional claims in maps and in any institutional affiliations.

## Supporting information


**Dataset S1** Dataset used for statistical analysis.

**Fig. S1** Illustration of the ten cultivars used in the experiment.
**Fig. S2** Schematics of the canopy traits measured at harvest.
**Fig. S3** Illustration of zone used for the quantity of motion analysis.
**Fig. S4** Density plot of wind speed and quantity of motion.
**Fig. S5** Additional correlations between windfleck properties and canopy structure.
Please note: Wiley is not responsible for the content or functionality of any Supporting Information supplied by the authors. Any queries (other than missing material) should be directed to the *New Phytologist* Central Office.

## Data Availability

The data that support the findings of this study are available in the Supporting Information of this article (Dataset S1).
