# Supplementary material for: When wuthering winds create fluttering fields: structural and biomechanical properties determine canopy light fluctuation properties of 10 wheat cultivars
Source: New Phytol. 2026 Feb 5;250(2):934–48. doi: 10.1111/nph.70975 (PMC13000972; doi:10.1111/nph.70975)

Supporting Information

Article title: When wuthering winds create fluttering fields: structural and biomechanical properties determine canopy light fluctuation properties of ten wheat cultivars.

Authors: Maxime Durand, Jonathon A. Gibbs, Erik H. Murchie, T. Matthew Robson, and Alexandra J. Gibbs

Article acceptance date: 13 January 2026

The following Supporting Information is available for this article:

Dataset S1: Dataset used for statistical analysis (see separate Excel file).

Figure S1: Illustration of the ten cultivars used in the experiment.

Figure S2: Schematics of the canopy traits measured at harvest.

Figure S3: Illustration of zone used for the quantity of motion analysis.

Figure S4: Density plot of wind speed and quantity of motion.

Figure S5: Additional correlations between windfleck properties and canopy structure.

Figure S1: Illustration of the ten cultivars used in the experiment. (a) Crusoe, (b) KWS Extase, (c) KWS Firefly, (d) KWS Kinetic, (e) Mayflower, (f) RGT Wilkinson, (g) Skyfall, (h) SY Insitor, (i) Theodore, and (j) Zoom.

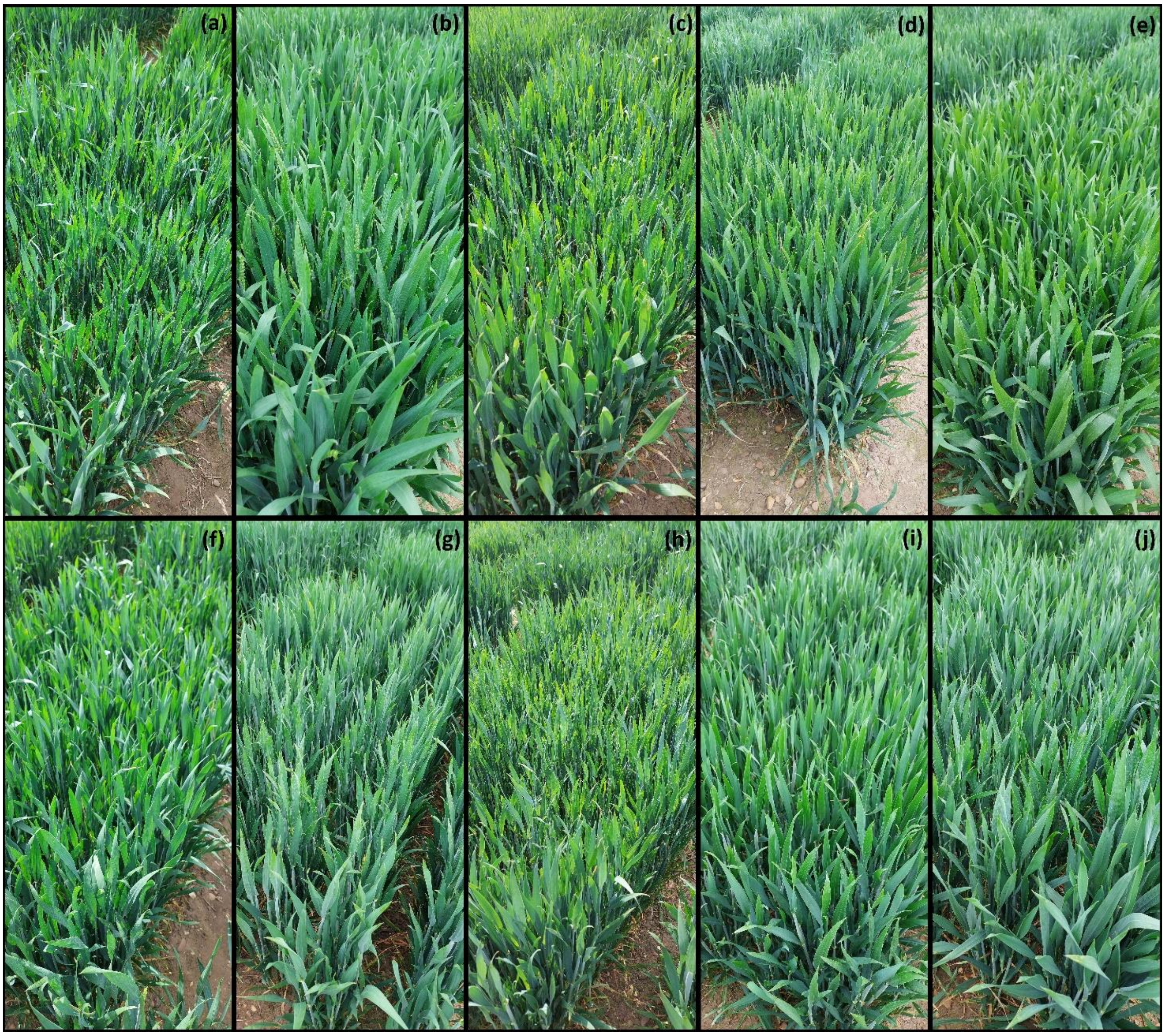

Figure S2: Schematics of the canopy traits measured at harvest.

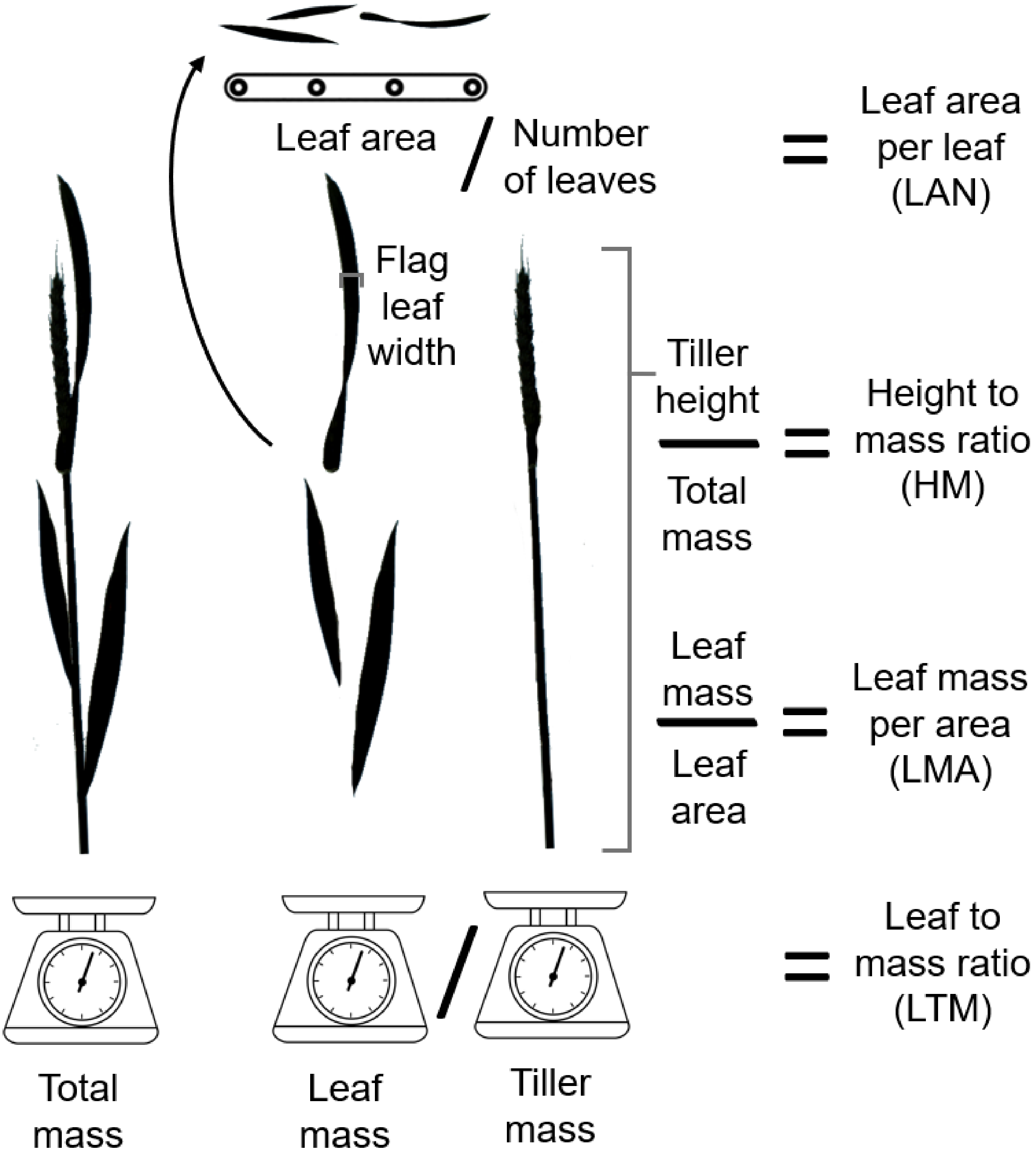

Figure S3. Illustration of zone used for the quantity of motion analysis. Top: first frame of the video used to map the measurement area (red rectangle) and the three reference zones (R1-R3, blue rectangles). The measurement area is further divided into a 16x9 grid and calculation are performed for a set of 18 zones. Down: Graphical representation of the 16x9 grid and each zone used. The gray area represents the border which was excluded from the analysis. Then, frame differencing was calculated for each of the 18 3x3 zones (9 in the blue and green area respectively). Black and red squares as well as light and darker shade of blue and green are displayed to highlight the 9 overlapping 3x3 square on each side.

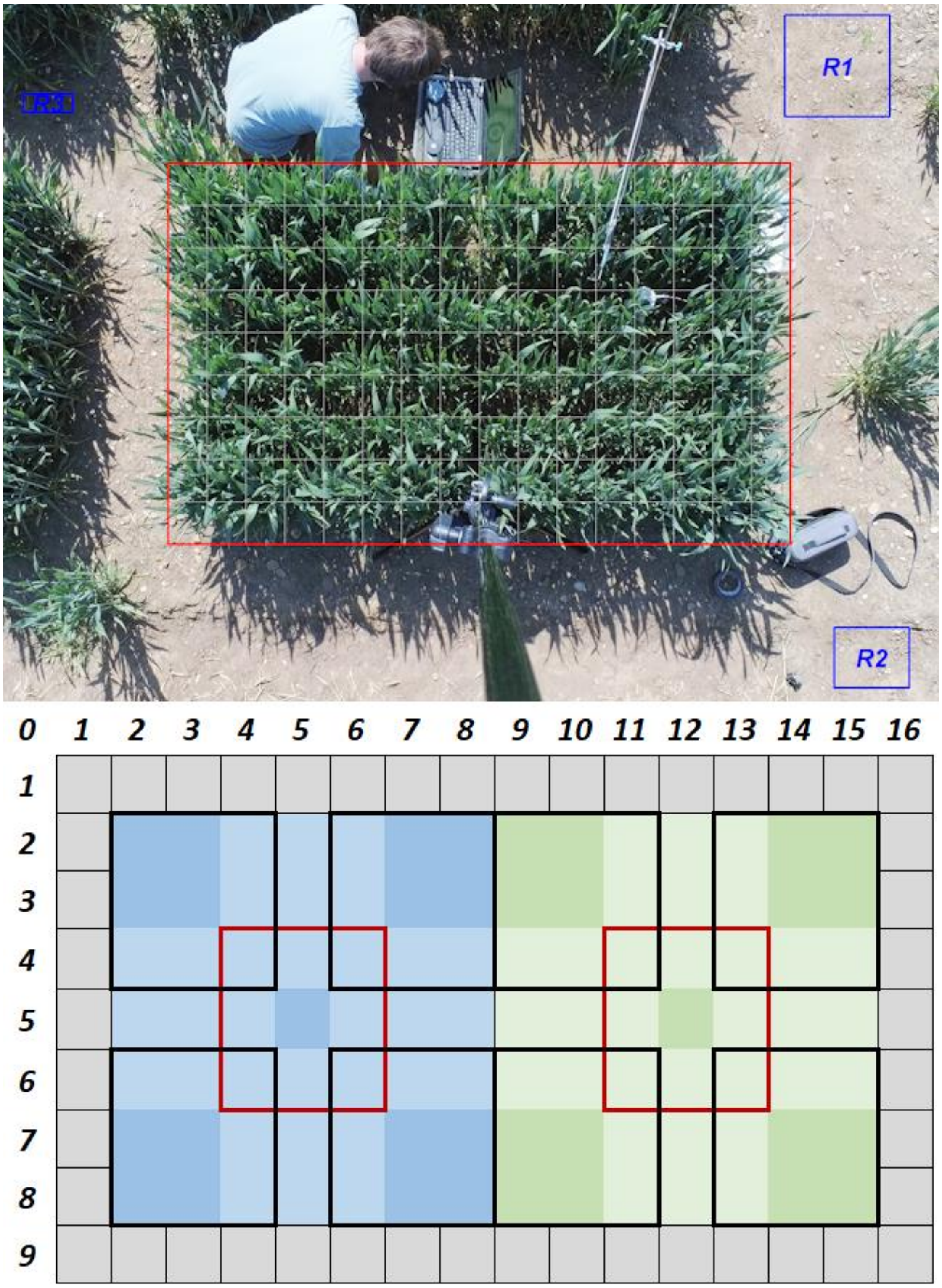

Figure S4. Density plot of wind speed and quantity of motion. (a) Density distribution of the quantity of motion calculated from the frame difference video analysis method. (b) Density distribution of wind speed recorded 1 m above the ground, over ten *Triticum aestivum* cultivars grown in the field at Nottingham, UK.

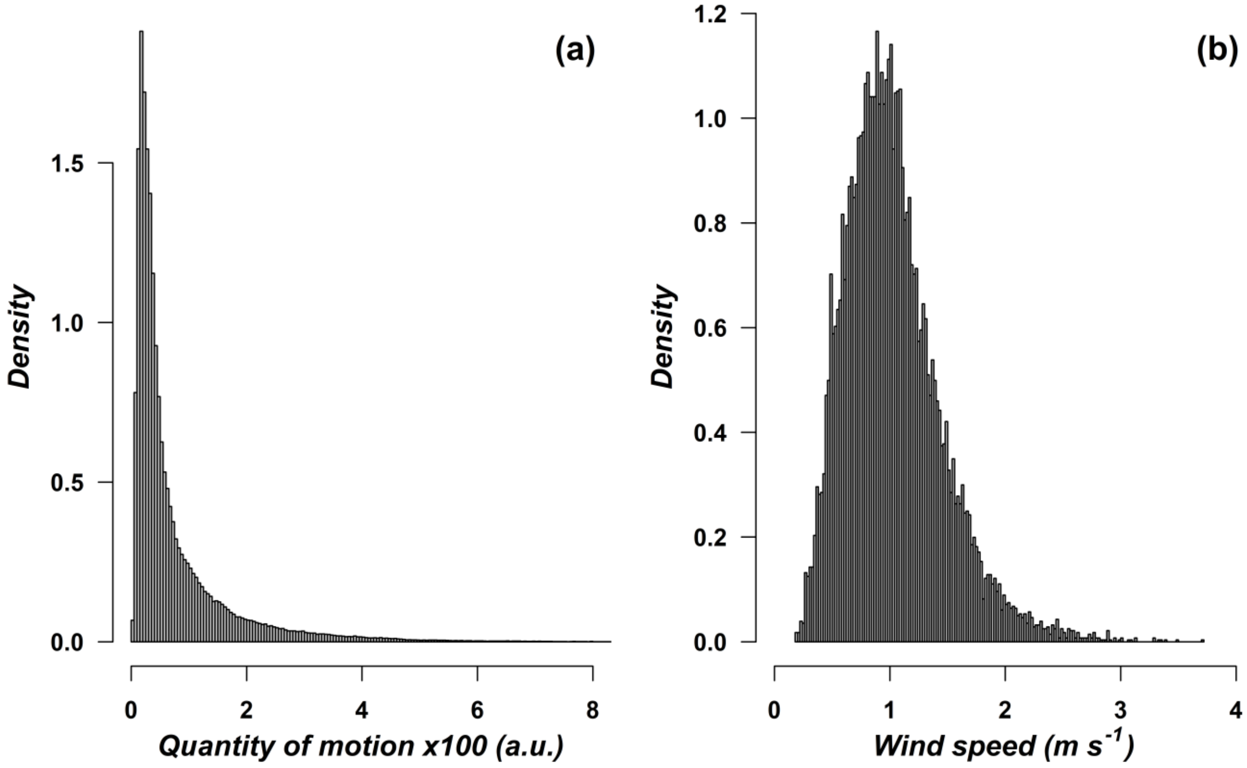

Figure S5. Additional correlations between windfleck properties and canopy structure in ten *Triticum aestivum* cultivars grown in the field at Nottingham, UK. *P* values of the linear regression are shown. Values are median ± standard errors after the block effect were accounted for by adding the residuals of a linear model with the block as main effect to the global mean.

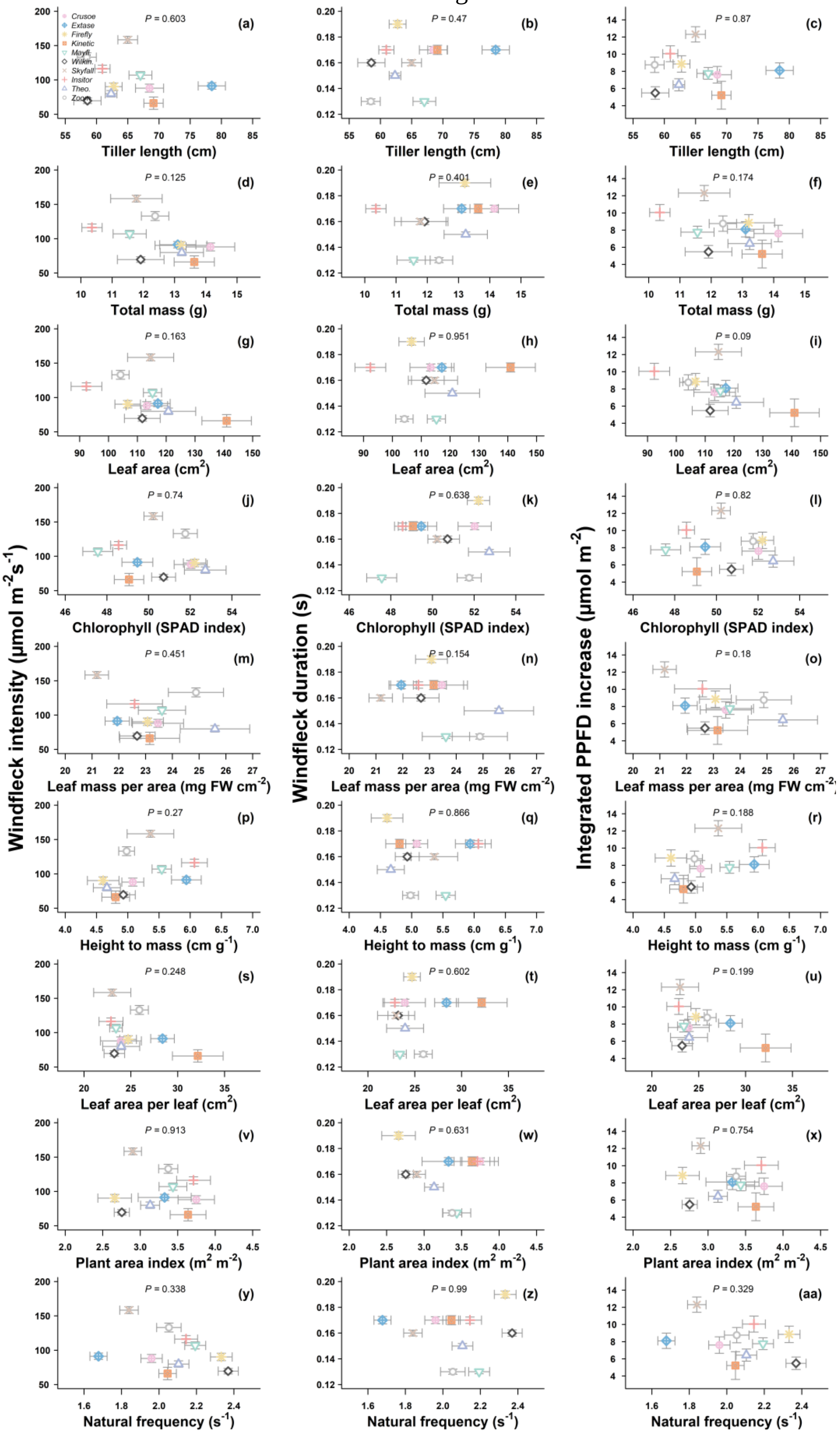

Supplement: Supplementary file 2 — Fig. S1 Illustration of the ten cultivars used in the experiment. Fig. S2 Schematics of the canopy traits measured at harvest. Fig. S3 Illustration of zone used for the quantity of motion analysis. Fig. S4 Density plot of wind speed and quantity of motion. Fig. S5 Additional correlations between windfleck properties and canopy structure. Please note: Wiley is not responsible for the content or functionality of any Supporting Information supplied by the authors. Any queries (other than missing material) should be directed to the New Phytologist Central Office. [file NPH-250-934-s002.pdf]
